# Our New York Letter

**Published:** 1893-01

**Authors:** 


### NEW YORK LETTER.

New York, Dec., 1892.

At a recent meeting of the section in obstetrics and gynecology at the Academy of Medicine, Dr. B. H. Wells related a case of adhesion of the nymphæ or labia-minora, some time after child-birth. The woman presented herself complaining of eczema of the ear, and gave no history that would lead to a vaginal examination until about to leave the office, when she made some remark about trouble with her husband, which lead the doctor to suspect that there might be some local trouble to account for her run-down condition beside the eczema. On examination the nymphæ were found so closly adherent that sexual intercourse was impossible though there was a small opening through which the menstrual discharges escaped so that the woman had not suspected that there was anything wrong with her genitals. The case was not only interesting on account of its rarity, but served to show the necessity of taking a thorough history of every case, however insignificant their trouble may seem to be.

Dr. J. C. Edger presented an apparatus for introducing sterilized glycerine into the uterus for the purpose of inducing labor in cases of deformity of the pelvis or other conditions demanding premature delivery. The apparatus consisted in a small glass syringe and rubber catheter, the end of which, beyond the eye, was filled with iodoform and plaster-of-paris to avoid the danger of introducing air into the uterus. Both of these and a bottle of glycerine can be readily sterilized at any house. The syringe is filled and a little forced out, to be sure there is no air left in the instrument, and the catheter passed well up into the uterine cavity, high enough to prevent the glycerine flowing out, and about half an ounce injected. The advantages claimed for this method over the old elastic catheter, are the perfect sterility that can be easily obtained, the rapidity with which it works, and the fact that it has never failed in any case. Dr. Edgar had only had occasion to use it in one case in which labor pains began within two hours after introducing the glycerine. Though this method is but little used in America at present, it is largely used in Europe, and from there we have the most pleasing report.

It seems to me that this method would be of great advantage to one practicing in the country where, when it is found necessary to induce labor, instead of passing a catheter and riding a number of miles only to return the next day, and likely as not find no pains; the physician can introduce the glycerine and have the child delivered in less time than it would take him to ride home and back again. The means by which glycerine induces labor is not fully known, though it is supposed to be through its affinity for water.

Dr. H. J. Garrigues read a paper on "Reprehensible, Unnecessary and Necessary Antiseptic Midwifery," in which he strongly condemned the absurd extremes to which antisepsis is carried by some physicians in the practice of midwifery. One case he mentioned was that of a Frenchman who used a corrosive sublimate solution, 1 to 2,000, for washing out the vagina twice daily for several weeks before confinement. In the last week, however, he introduced an iodoform tampon.

But, while such extremes are unnecessary and reprehensible in midwifery, a certain degree of antisepsis, or rather perfect asepsis, should be observed in all cases of midwifery. The value of antisepsis in midwifery is shown by the great reduction in the death rate before and after its introduction into the hospitals. In the New York Maternity Hospital, during the eight years, 1875 to 1883, out of 3,504 women confined, 146 died. During the first year after the adoption of the new method, in 1883, out of 102 deliveries there were no deaths, and since 1883, out of 3,170 deliveries, there have been only 30 deaths. But there are certain conditions existing in the Maternity, which if altered, might reduce even this low mortality as has been the case in the Sloan Maternity, and it may interest our readers to hear the methods by which these results are obtained. From January 1st, 1888, to October 1st, 1890, there were 1,000 women confined at the Sloan Maternity, out of which number, only six died and only one from septicæmia, from which she was suffering when she entered the hospital in the second stage of labor.

It must be remembered that patients treated at hospitals are rarely clean, are often filthy, and therefore precautions are necessary that would be out of place in private practice. On entering the hospital, each patient is given a full bath, plenty of soap being used, and special attention to the hair, which is thoroughly shampooed with delphinium or ether, or if very dirty, with a bichloride solution. A vaginal douche of a solution of corrosive sublimate of a strength of 1 to 5000, and a rectal enema of soap-suds are given. The woman is then attired in clean clothes, the property of the hospital, and placed in a special ward to await confinement. All confinements take place in a room separate from the wards which contains a table five feet long and three feet high with screw arrangement for elevating the foot in case of hemorrhage. The bedding is protected by a water-proof paper which forms an excellent substitute for the rubber sheet, and as it is inexpensive, is destroyed when soiled. When a patient is taken in labor, she is transferred to the delivery room where a vaginal douche and rectal enema are given early in the first stage. Physicians and nurses exercise the most scrupulous care with regard to cleanliness and disinfection. Before making a vaginal examination, the hands are scrubbed with a nail-brush; they are then immersed in alcohol and afterwards in a solution of bichloride, 1 to 2000.

Fifteen minutes after delivery, the placenta is expressed by the Crede method: A teaspoonful of the fluid extract of ergot is then administered; a vaginal douche of three pints of a bichloride solution 1 to 5000 at a temperature of 116 degs. F. is given, and the uterus held for one hour, when, if well contracted, the binder is applied. Not only is a binder applied to the abdomen but it extends from several inches below the trochanters to above the shoulders, thus holding the breasts firm. The breast binder is opened every two hours to let the child nurse. Women who have been confined before and not used the breast-binder, say it gives them more comfort than they had any idea could be had while in that state. An intra-uterine douche is only given in cases of instrumental delivery or when the hand has been introduced into the uterus. The entrance to the genital canal is closed by an antiseptic pad, twenty-eight inches long and eight inches wide, made of gauze and filled with absorbent cotton. On the first day, these pads are changed every four hours; on subsequent days, once in eight hours, the pads being smaller as the lochial discharge diminishes in quantity.

On the sixth day the patient is wrapped in a blanket and allowed to sit up for two hours; for five or six on the seventh and eighth days. On the ninth day the binder is removed and the patient allowed to walk about the wards, and on the tenth day, if all has gone well, she is discharged.

At a meeting of the New York Pathological Society, Dr. Hodenphyl presented a specimen showing miliary tubercles and gummata in the same lung. He spoke of the difficulty of making a diagnosis between tubercular and syphilitic inflammation and tumors, and formulated the following rules, which, though not conclusive, will aid in the diagnosis:

1. The history of syphilis or tuberculosis.

2. The bacteriological examination is usually positive in tuberculosis unless the tubercles are very old and fibrous: it is negative in syphilis.

3. Inoculation experiments are usually positive in tuberculosis and negative in syphilis.

4. Tubercles are usually present in large numbers and are scattered throughout the organ and throughout the body; gummatous deposits are usually single or in small numbers, and are confined to a single organ.

5. Tubercles are usually smaller than gummata.

6. Tubercles show a marked tendency to undergo cheesy degenerations and to form cavities; gummata rarely become cheesy, usually drying up and undergoing calcification.

7. Amyloid degeneration is usually present in tubercle, but, so far as known, is absent in gummata. On section, the tubercles appear as round grayish masses, often soft or cheesy in the centre, and surrounded by a zone of inflammatory products.

8. Microscopical examination will usually show the presence of tubercle bacillus in tubercles; but no characteristic micro-organism is present in syphilis. Giant cells are present usually in tuberculosis and are usually absent in syphilis.

